# Pairing limb posture feedback with an ankle exoskeleton to augment limb propulsion

**DOI:** 10.1371/journal.pone.0335054

**Published:** 2025-10-22

**Authors:** Steven A. Thompson, Emily E. Foley, Jason R. Franz, Gregory S. Sawicki, Michael D. Lewek

**Affiliations:** 1 Lampe Joint Department of Biomedical Engineering, University of North Carolina at Chapel Hill and North Carolina State University, Chapel Hill, North Carolina, United States of America; 2 George W. Woodruff School of Mechanical Engineering, Georgia Institute of Technology, Atlanta, Georgia, United States of America; 3 Division of Physical Therapy, Department of Health Sciences, University of North Carolina at Chapel Hill, Chapel Hill, North Carolina, United States of America; University of Illinois Urbana-Champaign, UNITED STATES OF AMERICA

## Abstract

Limb propulsion deficits are common in a variety of clinical populations and may arise from a decreased plantarflexor moment and/or decreased trailing limb angle (TLA). Ankle exoskeletons (EXOs) can augment plantarflexor moment, but observations of a concurrently reduced TLA has limited the conversion of increased plantarflexor moment to increased propulsion in people following stroke. The purpose of this study was to assess the interaction of unilateral EXO (plantarflexor) assistance and TLA feedback on gait propulsion, joint mechanics, and margins of stability. Ten young, unimpaired individuals walked on an instrumented treadmill with the EXO worn but unpowered to obtain baseline peak TLA. Participants then walked with visual feedback of TLA while attempting to match a target peak TLA of baseline-5°, baseline, or baseline+5° in random order. For each target TLA, participants walked with three EXO plantarflexor torque magnitudes, with peak assistance proportional to bodyweight (0%, 15%, and 35% bodyweight) in random order. Propulsive impulse significantly increased as peak TLA increased but was not affected by EXO assistance. Higher EXO assistance resulted in a proximal-to-distal shift in positive mechanical work performed by the lower limb, observed as an increase in the relative contribution of the ankle to total ankle and hip positive mechanical work. Increased TLA significantly reduced the magnitude of the anteroposterior margin of stability at toe-off, while higher EXO assistance yielded larger anteroposterior margins of stability. Margins of stability in the mediolateral direction were not affected by TLA or EXO assistance. This study highlights the potential for increasing propulsion through feedback of TLA, but with potential negative impacts to stability. For populations with deficits in plantarflexor moment, the use of visual feedback to prevent a reduction in TLA while walking with ankle EXOs may allow conversion of joint benefits to benefits in limb propulsion.

## Introduction

The ability to walk can influence participation in daily activities [1,2] and impact quality of life [3]. Walking involves several biomechanical subtasks, including vertical support, lateral stability, swing limb advancement, and forward propulsion [4]. Limb propulsion deficits are especially common with aging [5–7] and following neurological injury and disease [8]. Reductions in propulsion are associated with reduced gait speed [8], increased energy cost [9], and decreased endurance [10], making limb propulsion deficits a potentially relevant rehabilitation target. Operationally derived from the anterior component of the ground reaction force (GRF), limb propulsion is primarily a function of both the ankle plantarflexor moment and the trailing limb angle (TLA) [11–13]. Consequently, the plantarflexor moment and/or the trailing limb angle can be targeted to theoretically increase limb propulsion.

The use of an ankle exoskeleton (EXO) can overcome an inadequate ankle plantarflexor moment [14,15] and power [14]. However, the use of EXOs has not consistently converted those local ankle joint benefits into enhanced propulsion and gait performance in people post-stroke [14,15]. Instead, people post-stroke walking with an ankle EXO appear to reduce the TLA such that any increase in plantarflexor moment from the EXO is not translated to increased propulsion [14,15]. Importantly, people with propulsion deficits have the capability to increase TLA, and consequently limb propulsion [16]. Thus, determining ways to increase or prevent a decrease in TLA while walking with an ankle EXO is important for enhancing gait in these populations.

The inherent ability to increase TLA in those with propulsion deficits suggests that the use of visual feedback of TLA may enhance propulsion while walking with an ankle EXO. In fact, real-time feedback has successfully augmented propulsion through instruction of propulsive forces [17–21] or joint kinematics [19,20,22–24]. With the use of an ankle EXO to augment plantarflexor moments, the addition of TLA feedback may provide a solution to prevent the instinctive reduction in TLA and thus allow EXO-induced increases in limb propulsion [11,12,19,20]. Although the feasibility of combining an ankle EXO with TLA feedback has recently been explored in individuals with chronic stroke [25], the interaction between ankle EXO torque and TLA feedback on limb- and joint-level biomechanics remains unexplored.

Manipulating ankle joint kinetics with EXO assistance and hip joint kinetics with TLA feedback may cause changes in mechanical work performed by these joints in the lower limb. In fact, mechanical work performed by the lower limb is commonly redistributed across joints in ageing populations [5,26] and populations with neurologic conditions [27–29]. Specifically, a distal-to-proximal redistribution may arise from the need to compensate for more affected distal (i.e., ankle) muscles with less affected proximal (i.e., hip) musculature. Thus, it is important to investigate how ankle and hip kinetics, especially their relationship, are altered when manipulating these joints with EXO assistance and kinematic feedback during walking.

It is possible that increasing TLA might decrease walking stability because increasing TLA while maintaining walking speed is associated with longer step lengths and reduced cadence [30]. Walking with step lengths longer than typical and cadences slower than typical have been associated with less stability as evidenced by smaller margins of stability (MoS) [31–33]. It is therefore necessary to investigate potential deficits to stability with increasing TLA. Although incompletely understood, the MoS may provide insight into stability while walking [34,35].

Given the need to understand how people adjust relevant gait parameters (i.e., TLA) while walking with an ankle EXO, the primary purpose of this study was to assess the combined effect of pairing visual feedback of TLA with a powered ankle EXO on limb propulsion in young, unimpaired individuals. Secondarily, we assessed how various combinations of TLA magnitude, and peak EXO torque magnitude affected the distribution of positive mechanical work performed by joints in the lower limb. Finally, we investigated how MoS_ML_ and MoS_AP_ at toe-off of the assisted (i.e., test) limb change with the use of ankle EXOs and feedback of TLA to aid interpretations and future research in clinical populations. Before investigating the mechanics associated with ankle EXO assistance and TLA feedback in clinical populations, it is necessary to assess these interventions in young, unimpaired populations to isolate gait changes caused by the interventions themselves, without the confounding factors of a disordered neuromuscular system. With young, unimpaired participants walking at a fixed speed, we hypothesized that propulsion would not increase with higher EXO assistance levels when TLA is decreased and that increasing TLA would yield increased propulsion, independent of EXO torque. We also hypothesized that increasing TLA would cause a transfer of work from the ankle to the hip, because ankle moment and power requirements may be reduced as TLA increases at a fixed speed. Finally, we hypothesized that greater peak TLA would reduce both MoS_ML_ [32] and MoS_AP_ [31–33] at test limb toe-off, considering the relationship between TLA and spatiotemporal characteristics of gait [30].

## Materials and methods

### Participants

Ten young, unimpaired adults provided written, informed consent prior to participation in this study. This study was approved by the University of North Carolina at Chapel Hill’s Institutional Review Board (study #21–0929). The recruitment period for this study started on 30 October 2023 and ended on 26 January 2024. Participants were included if they reported being able to walk uninterrupted for 20 minutes without assistance and without self-reported uncontrolled cardiovascular, respiratory, or metabolic problems. Additionally, participants were excluded if they self-reported orthopedic or neurological disorders that may alter gait mechanics or a history of balance deficits or uncontrolled seizures.

### Study design

**Fig 1:**
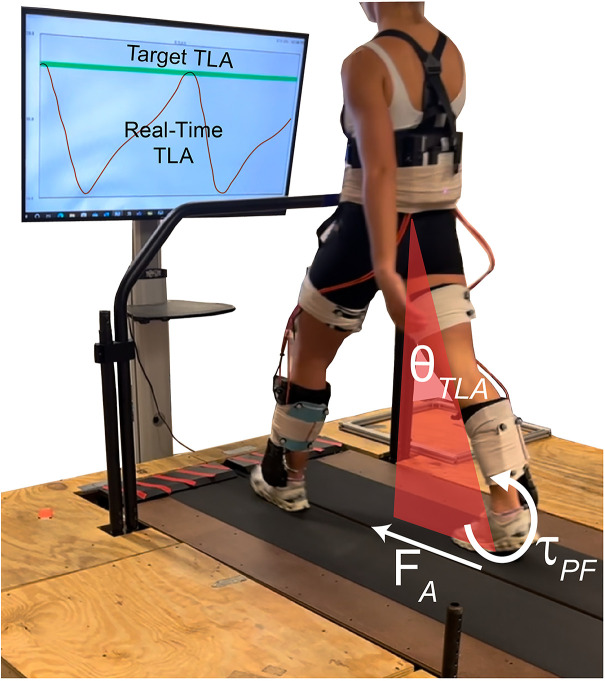
Feedback of trailing limb angle. Trailing limb angle (θ_TLA_) and plantarflexor moment (τ_PF_) contributing to propulsion (F_A_). Real-time feedback of TLA (red) and target TLA (green) displayed on a TV monitor in front of participants walking on a treadmill.

**Fig 2:**
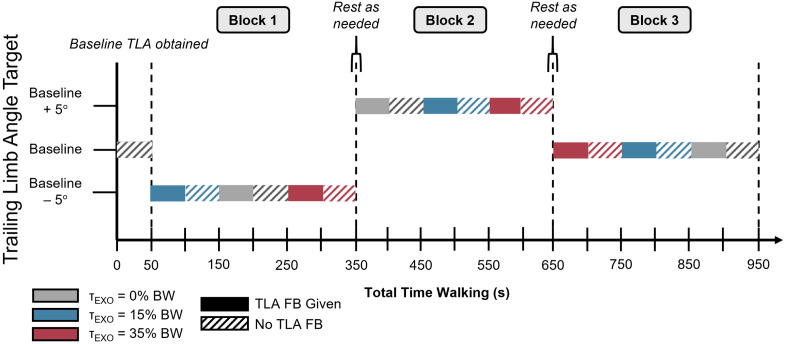
Example protocol of a testing session. TLA is denoted on the y-axis and the color of each bar denotes the imposed peak exoskeleton torque. Baseline TLA was first obtained, and target TLA was then block order randomized. Order of exoskeleton torque was randomized within each TLA block to obtain 9 combinations of target TLA and prescribed exoskeleton torque. Only time walking on the treadmill is shown, but participants rested as needed between TLA conditions. After 50 seconds of walking with visual feedback (TLA FB Given), visual feedback was removed as participants continued walking on the treadmill (No TLA FB) for a brief washout.

For all testing, participants walked on a dual-belt instrumented treadmill (Bertec, Columbus, OH) at 1.0 m/s, consistent with what is expected for many clinical populations [36], while wearing a bilateral powered ankle EXO (Biomotum, Portland, OR) [37–39]. During powered conditions, plantarflexor torque assistance was provided to one limb only (referred to below as the test limb) with proportional estimated joint moment control based on bodyweight (BW) [37]. First, participants’ baseline TLA (Fig 1) was obtained for the test limb from a 50-s walking trial, while wearing the EXO in an unpowered state. After the baseline condition, we used a block-randomized design to provide real-time visual feedback of test limb peak TLA. The target TLA was randomized to either baseline TLA, baseline+5°, or baseline-5°. During each of the three target TLAs, participants walked with randomized levels of EXO assistance (0%, 15%, and 35% BW plantarflexor torque) for a total of 9 experimental conditions. Following a brief acclimation period (~10 s), each trial lasted 50 s. Participants were allowed to rest as needed (~5 mins.) between target TLA conditions. We provide an example protocol in Fig 2. We collected kinematic data at 120 Hz using marker-based motion capture (Vicon, Los Angeles, CA) and synchronized GRFs at 960 Hz from the treadmill. Prior to walking, retroreflective markers were placed bilaterally on the second metatarsal head, fifth metatarsal head, center of the posterior calcaneus, medial and lateral malleoli, medial and lateral femoral condyles, greater trochanter, and iliac crest. Clusters of four markers were placed on the posterior aspect of each shank and thigh and a three-marker cluster was placed on the anterior pelvis to accommodate the EXO motor that rested on the posterior pelvis.

### Real-time feedback

Test limb TLA was measured in real-time (Visual3D, C-Motion) by calculating the angle between the laboratory’s vertical axis and a line connecting the markers placed on the greater trochanter and the 5^th^ metatarsal head [11,12,40]. This angle was displayed continuously on a TV monitor in front of the participants as they walked (Fig 1). Target TLA was displayed as a green band with a ± 1° height, centered at the target, and participants were instructed to reach the target band with each step. Participants were able to practice with the feedback while standing and by walking prior to beginning a condition to ensure they understood how their movement affected TLA in real-time.

### Data processing

GRFs were processed using a 4^th^ order lowpass Butterworth filter with a cutoff frequency of 20 Hz and normalized to the participants’ BW. Propulsive impulse was computed as the time integral of the positive portion of the anteroposterior component of the GRF vector over each gait cycle [41]. Propulsive impulse was then averaged across all available gait cycles for each condition for each participant.

Marker trajectories were processed using a 4^th^ order lowpass Butterworth filter with a cutoff frequency of 6 Hz and joint angles were calculated using Euler angles for the ankle, knee, and hip. Inverse dynamics were used to compute joint moments (Visual 3D, C-Motion), which were normalized to participants’ mass. TLA was computed as previously described [11,12,40] and peak TLA was averaged across gait cycles for each condition for each participant. Peak TLA was then normalized by subtracting participants’ baseline peak, such that TLA is reported as the difference from baseline. Mechanical power was calculated for each joint as the product of joint angular velocity and moment. Positive mechanical work for each joint was computed as the time integral of the positive portion of the mechanical power curve and was averaged across gait cycles for each condition for each participant. At the ankle, biological moment and mechanical power were calculated by subtracting the EXO torque and power from the total calculated with motion capture and GRFs (i.e., biological = total – EXO). Total mechanical work was computed as the sum of mechanical work performed by the total ankle ($W_{A}^{+}$), knee ($W_{K}^{+}$), and hip ($W_{H}^{+}$). To assess the relative distribution of positive mechanical work by each joint in the lower limb, we calculated the fraction of each joint’s positive mechanical work to the total positive mechanical work. To assess the redistribution of positive mechanical work between the ankle and the hip, we calculated the redistribution ratio (RR) between the ankle and the hip for each condition, where $RR=\text{1}-\;\frac{\left(W_{A}^{+}-\;W_{H}^{+}\right)}{{(W}_{A}^{+}+\;W_{H}^{+})}$ [42]. A ratio of 0 indicates that all the positive work from these two joints was done by the ankle, whereas a ratio of 2 indicates that all the positive work was performed by the hip.

MoS was calculated in accordance with the conventions described by [34]. The center of mass (CoM) location was determined using GRFs [34,43–45]. The extrapolated CoM (XcoM) was then calculated by adding the estimated CoM to the velocity of the CoM divided by the eigenfrequency of the pendulum (ω_0_), where ω_0_ = $\sqrt{g/l}$, given *g* = gravity and l = effective pendulum len*g*th. Treadmill velocity was accounted for in the anteroposterior direction [34]. MoS was then calculated as the base of support (BoS) position – XcoM in each direction using the net CoP location as the BoS during walking [34]. MoS is reported so that a positive MoS_ML_ indicates the BoS is lateral to the XcoM in the mediolateral direction and a negative MoS_AP_ indicates the BoS is posterior to the XcoM [34]. Because our experimental conditions (TLA and EXO torque magnitude) are most focused on the instant of test limb toe-off, we were primarily concerned with MoS at the instant of test limb toe-off, where the BoS is most dominated by the non-test limb. Importantly, the MoS is generally at a minimum around the instant of contralateral toe-off [34,35,46]. Nevertheless, MoS was calculated separately for the test and non-test limb due to possible asymmetry associated with unilateral EXO assistance and TLA feedback.

### Statistical analysis

Statistical analyses were completed in SPSS (v26, IBM, Armonk, NY). Peak TLA, propulsive impulse, RR, and MoS_ML_ and MoS_AP_ at the instant of test limb toe-off were computed for the test limb and compared across conditions with a 2-way (TLA x EXO torque) repeated measures ANOVA. Paired t-tests were used as post-hoc tests with a Bonferroni correction for multiple comparisons. Data were lost completely at random [47,48] for one condition for one participant (target TLA = baseline-5°, imposed EXO torque = 15% BW) requiring multiple imputation [47–49] using a MATLAB custom function developed by Folch-Fortuny et al. [50].

## Results

### Participants

Of the 10 participants recruited (5 female, 5 male), two participants did not receive the intended EXO assistance parameters because of device malfunction and thus were excluded from analysis due to failure to complete the full protocol. Data from the remaining 8 participants (3 female, 5 male) were used for analysis. Participants were (mean±SD) 21.0 ± 1.8 years old, 1.76 ± 0.12 m tall, and had a mass of 71.0 ± 17.7 kg.

### Peak trailing limb angle

**Fig 3:**
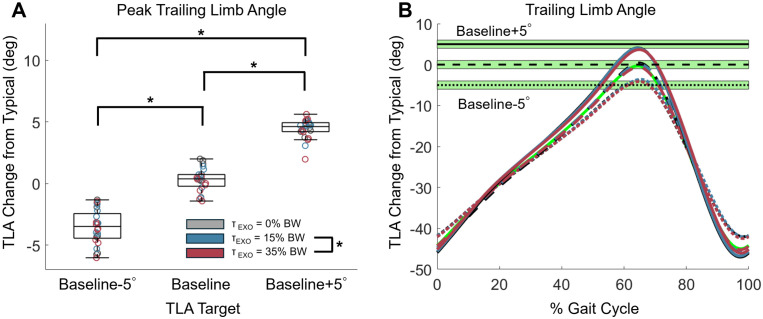
Peak trailing limb angle. (A) Peak TLA for each target TLA condition, relative to typical peak, for each participant. Individual participant data are circles, with color denoting the imposed peak exoskeleton torque magnitude. *p < 0.05. (B) Time series of TLA, relative to typical peak, throughout the gait cycle for the three target conditions (green horizontal bands). Curves are averaged across participants for each of the conditions. Target TLA of baseline+5° is denoted by solid lines, target of the baseline TLA is denoted by dashed lines, and target TLA of baseline-5° is denoted by dotted lines. Exoskeleton peak torque magnitude is denoted by color. The green, dashed TLA curve is TLA during the condition for obtaining baseline.

All participants were able to adjust their peak TLA in response to our real-time feedback while the EXO was both unpowered and powered. Baseline peak TLA was 20.9 ± 1.3°. By design, we observed a main effect of TLA feedback condition on peak TLA (p < 0.001, η^2^_p_=0.973) (Fig 3A). When the baseline TLA was prescribed as the target, peak TLA difference from baseline was 0.30 ± 0.95° which falls within the target band (Fig 3B). For the −5° TLA target condition, peak TLA was significantly less than baseline (−3.55 ± 1.40°, p < 0.001). Peak TLA was significantly greater than baseline with the + 5° TLA target (+4.42 ± 0.79°, p < 0.001). Changing the EXO torque magnitude also had a main effect on peak TLA (p = 0.01, η^2^_p_=0.484), with a 0.52° smaller peak TLA on average in the 35% BW EXO conditions compared to 15% BW EXO conditions (p = 0.014), but no difference between any other conditions (p ≥ 0.101). No interaction effect of EXO assistance magnitude and target TLA was observed (p = 0.334, η^2^_p_=0.146).

### Limb propulsion

**Fig 4:**
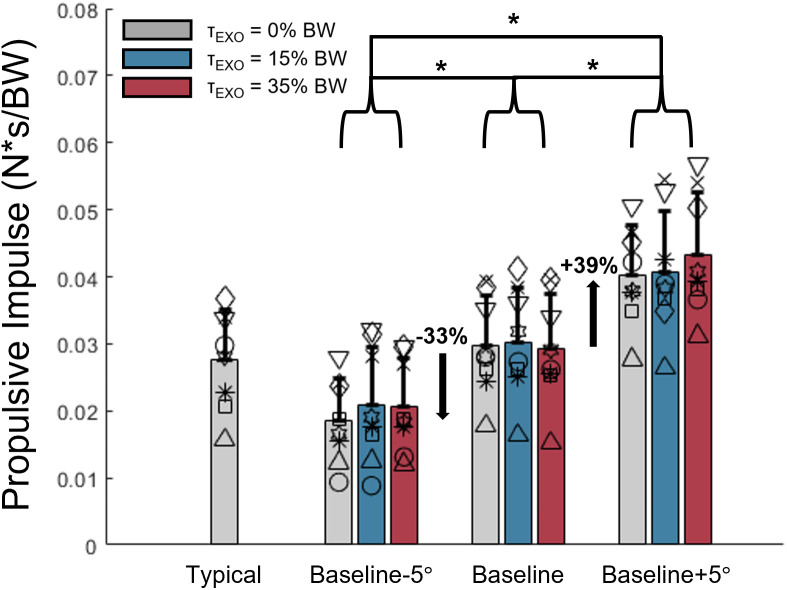
Limb propulsion. Propulsive impulse for each combination of target TLA and imposed peak exoskeleton torque magnitude. Percent differences are reported with respect to the condition where participants’ baseline TLA was the target. *p < 0.05.

We observed no interaction effect between target TLA and EXO torque magnitude on propulsive impulse (p = 0.174, η^2^_p_=0.197), but did note that both TLA (p < 0.001, η^2^_p_=0.933) and EXO torque magnitude (p = 0.042, η^2^_p_=0.364) had main effects on propulsive impulse (Fig 4). When targeting baseline peak TLA, propulsive impulse was 0.030 ± 0.007 N*s/BW. Relative to this value, propulsive impulse decreased when targeting the smaller TLA (i.e., −33%, 0.020 ± 0.007 N*s/BW, p = 0.001, 95% CI [−0.014, −0.005]) and increased when targeting the larger TLA (+39%, i.e., 0.041 ± 0.008 N*s/BW, p < 0.001, 95% CI [0.007, 0.016]). As such, propulsive impulse was greater when targeting the larger TLA than when targeting the smaller TLA (p < 0.001, 95% CI [0.016, 0.027]). Despite a significant main effect for EXO torque, post-hoc analysis revealed no significant pairwise difference in propulsive impulse. Average propulsive impulse were 0.030 ± 0.011 N*s/BW, 0.030 ± 0.012 N*s/BW, and 0.031 ± 0.012 N*s/BW for the 0%, 15%, and 35% EXO torque magnitude conditions, respectively (all p ≥ 0.084) across all TLA targets. Change in propulsive impulse from the 0% EXO condition to the 15% and 35% EXO conditions for each of the target TLA conditions is provided in the Supporting Information (S1 Fig) to aid interpretation of the effects of EXO assistance on propulsion.

### Joint mechanics

**Fig 5:**
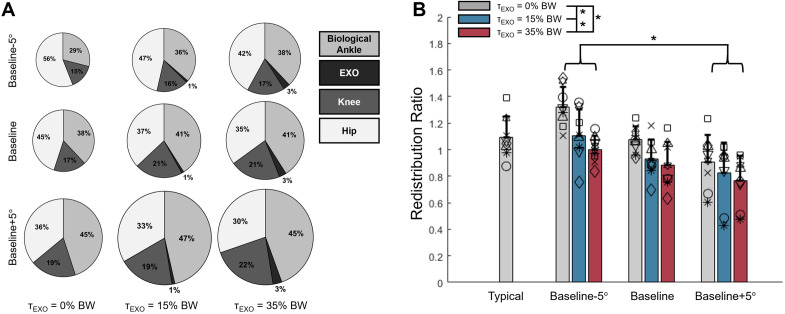
Joint-level positive mechanical work. (A) Joint contributions to the total positive mechanical work performed by the test limb over a stride. The total amount of positive mechanical work performed by the test limb over a stride is proportional to the radius of the pie chart. (B) Redistribution ratio of positive mechanical work performed by the ankle and hip over a stride *p < 0.05.

For all conditions, the total positive mechanical work performed by the lower limb ranged from 0.76 J/kg to 1.09 J/kg on average over a stride (Fig 5A). We observed the least total positive mechanical work for the −5° TLA, unpowered EXO condition and the most for the + 5° TLA, 35% BW peak EXO torque condition. The magnitude of positive mechanical work performed by each joint of the test limb and the EXO is provided in the Supporting Information (S1 Table). Main effects of both TLA (p = 0.002, η^2^_p_=0.582) and imposed EXO torque magnitude (p = 0.001, η^2^_p_=0.756) were observed on RR values (Fig 5B). RR was larger (i.e., relatively more positive mechanical work performed proximally) for the −5° TLA compared to the + 5° TLA conditions (p = 0.028). No other differences were observed between TLA conditions (all p ≥ 0.063). When the EXO was unpowered, RR was 1.10 ± 0.23, implying that the hip habitually performed slightly more positive mechanical work than the ankle. When the EXO was powered, the ankle performed more positive mechanical work than the hip. Specifically, RR was 0.95 ± 0.22 (p = 0.029 vs. unpowered) for 15% BW EXO assistance and 0.88 ± 0.18 for 35% BW EXO assistance (p = 0.002 vs. unpowered, p = 0.006 vs 15% BW condition). No interaction effects were observed for RR (p = 0.110, η^2^_p_=0.229).

### Margins of stability

**Fig 6:**
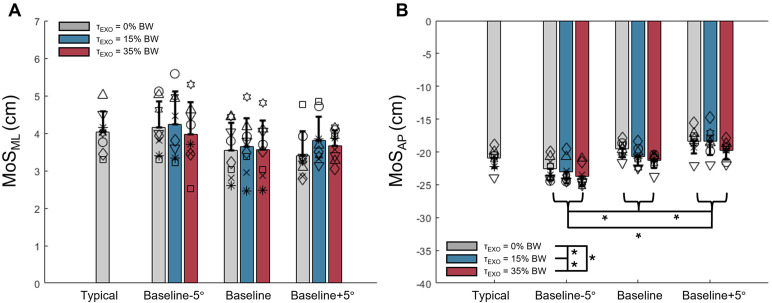
Margins of stability. (A) MoS_ML_ at the instant of test limb toe-off. Positive values indicate the base of support is lateral to the extrapolated center of mass (XCoM). *p < 0.05. (B) MoS_AP_ at the instant of test limb toe-off. Negative values indicate the base of support is posterior to the XCoM. *p < 0.05.

We observed no main effect of prescribed TLA (p = 0.159, η^2^_p_=0.231) or EXO torque magnitude (p = 0.297, η^2^_p_=0.159) on MoS_ML_ at test limb toe-off (Fig 6A). However, both target TLA (p < 0.001, η^2^_p_=0.860) and EXO torque (p < 0.001, η^2^_p_=0.738) had main effects on MoS_AP_ at test limb toe-off (Fig 6B). MoS_AP_ magnitude increased as target peak TLA decreased from baseline to baseline-5° (p = 0.003). MoS_AP_ magnitude was smallest during the baseline+5° peak TLA condition (p < 0.001 vs −5° TLA target, p = 0.024 vs baseline). Specifically, average MoS_AP_ magnitude during the baseline+5° TLA conditions was 2.16 cm smaller (i.e., less negative) than MoS_AP_ during typical walking. MoS_AP_ magnitude increased with each successive increase in EXO torque magnitude (all p ≤ 0.039). No interaction effects on MoS_ML_ (p = 0.162, η^2^_p_=0.202) or MoS_AP_ (p = 0.076, η^2^_p_=0.254) were observed. To aid interpretation of MoS data, step lengths and cadences are reported in the associated open-source data file.

## Discussion

Results from this study partially support our overall hypothesis that pairing visual feedback of TLA with an ankle EXO can enhance propulsion and increase the ankle joint’s relative contribution to positive mechanical work while walking on a treadmill. Specifically, we showed that participants were able to adjust TLA in response to real-time feedback while walking with a powered ankle EXO. Additionally, we showed that: (1) increasing TLA with feedback increased limb propulsion, independent of EXO torque, (2) EXO torque redistributed positive mechanical work from the hip to the ankle, and (3) increasing TLA with feedback reduced MoS_AP_ magnitude (i.e., more ‘unstable’), while higher levels of EXO torque increased MoS_AP_ magnitude (i.e., more ‘stable’).

Physical assistive devices, such as EXOs, are increasingly being used in humans’ daily lives for rehabilitation, performance enhancement, and injury prevention. To fully integrate humans with powered assistive devices, it is necessary to ensure people can benefit from the device. Much of the research in human-device interactions focuses on device design and control to improve integration, safety, and attachment dynamics of the device with the user [51–57]. Instead, we are focused on the user’s motor control to enhance symbiosis of the human-device relationship. For example, previous studies have demonstrated that local ankle joint benefits from EXO assistance alone does not result in enhanced limb propulsion [14,15], potentially due to a change in the user’s movement strategy as indicated by a reduction in TLA [14]. Our goal was to manipulate the user’s movement with real-time feedback, allowing for propulsion augmentation while walking with an ankle EXO.

Our results show that people without gait impairments can augment propulsion while walking with visual feedback intended to increase TLA. Therefore, our hypothesis that increasing TLA would yield increased propulsive impulse was supported. Although post-hoc analysis revealed no effect of EXO assistance on propulsive impulse, our participants were young, unimpaired adults without deficits in biological ankle moment and thus did not need EXO-induced augmentation. External cues forced changes in TLA, while ankle kinetics were not targeted. EXO assistance was free to compensate for, instead of supplement, the biological ankle moment. It is therefore not surprising that target TLA, but not plantarflexor assistance, affected propulsive impulse in this population. For populations with deficits in biological ankle moment, the combination of ankle EXO assistance and real-time feedback of TLA may offer a means to augment propulsion by enhancing the ankle plantarflexor moment [14,15] without users hindering these benefits through a reduced TLA [25].

We also observed that ankle plantarflexor assistance can redistribute positive mechanical work performed by lower limb joints, which has been shown in previous studies in individuals without impairment [58,59] and individuals post stroke [14]. Specifically, higher EXO assistance elicited a proximal-to-distal redistribution of positive mechanical work, from the hip to the ankle, compared to unassisted walking. This redistribution, observed here in young, unimpaired participants, has important implications for populations with gait impairment. For example, people with stroke tend to compensate for weakened distal muscles by performing more mechanical work using muscle-tendon units spanning the hip [27–29]. The results here, although from individuals without distal muscle weakness, offer potential for the use of EXOs to redistribute mechanical work back to the ankle, leading to reduced metabolic cost and increased limb propulsion during gait [60–62]. It is worth noting that participants here walked with a higher typical RR than reported by other authors who tested young, unimpaired adults [42,63]. We believe that this apparent discrepancy may be due to reduced propulsion or TLA [42] from participants walking at speeds slower than typical for this population [64], and closer to that of populations with gait impairment [36] where we expect greater proximal musculature contribution to positive mechanical work performed by the lower limb [27–29].

Our data support the need to continue evaluating gait stability outcomes with changes imposed on TLA. Specifically, our findings suggest that feedback to increase TLA has the potential to induce less stable walking, as evident by a smaller magnitude (i.e., less negative) MoS_AP_. The reduction in MoS_AP_ magnitude observed here does not necessarily indicate ‘unstable’ walking, however, as no participants reported feeling unstable and no falls occurred throughout the study. Consequently, these data compliment that of others who have reported lesser anteroposterior stability with longer steps (i.e., larger TLA) [31–33]. We speculate that decreasing TLA, and therefore step length, in some individuals with gait pathology may represent a mechanism by which individuals create greater stability. The fact that MoS_AP_ magnitude increased (i.e., more negative) with higher EXO assistance suggests that the EXO might partially offset any potential instability induced by the larger imposed TLA. Nevertheless, we cannot conclude whether this increased MoS was a result of EXO assistance itself or a subconscious response by the users as they responded to EXO assistance to protect stability. Regardless, our findings here suggest that anteroposterior stability measurements, such as the MoS_AP_, may be beneficially altered with ankle EXO assistance and detrimentally affected by feedback of larger TLA. Here, MoS_AP_ magnitude decreased (i.e., less negative) by an average of 2.16 cm during the baseline+5° TLA condition compared to typical walking. This magnitude of change in MoS_AP_ is comparable to changes reported by other studies that involved walking with perturbations [65] or with altered spatiotemporal aspects of gait [66]. Conversely, other studies have reported much larger changes in MoS_AP_ magnitude of up to 14 cm with perturbations [67] and up to 8.9 cm with changes in spatiotemporal aspects of gait [33]. Although the magnitude of changes in anteroposterior stability found here by increasing TLA are likely to be well accommodated by individuals with and without impairment, they should be considered when delivering and analyzing outcomes associated with these interventions. Future studies involving participants with compromised balance, such as those with stroke, may consider personalized TLA targets or adaptive feedback protocols if participants appear to be at increased risk of experiencing gait instability while walking with feedback of increased TLA.

The lack of changes in MoS_ML_ with TLA feedback and EXO assistance in this study is promising for implementing these interventions without hindering mediolateral stability. Here, we calculated the MoS at the instant of test -limb toe-off, where the magnitude of the MoS is near minimum [34,35,46] and when both TLA and EXO assistance magnitude are close to their peaks, creating the greatest potential for instability. MoS was calculated only at test limb toe-off because we provided unilateral, and therefore asymmetric, EXO assistance and feedback of TLA. It is possible that these interventions also have effects on stability at other points in the gait cycle and thus should be further investigated.

To target the decreased TLA observed when walking with EXO assistance [14], we provided feedback directly of TLA. To augment propulsion, other studies have provided feedback of propulsive forces, where participants have the freedom to explore solutions to reach the target including both increased peak ankle plantarflexor moment [18–20] and increased TLA [18–21]. Because ankle EXOs have been shown to increase plantarflexor moment [14,15], preventing a concomitant reduction in TLA using feedback may allow for propulsion augmentation without increasing energy demands, which may be required to increase the biological plantarflexor moment in populations with propulsion deficits. Additionally, TLA feedback may be both more feasible and cheaper to implement in overground walking than feedback regarding propulsive forces as it can be measured with inertial measurement units in real time [25,68]. Given our findings on varying levels of EXO assistance and TLA feedback levels on gait outcomes, coupled with the feasibility of using TLA feedback with walking with an ankle EXO in people post-stroke [25], future studies will examine the interaction between various levels of EXO assistance and TLA targets in clinical populations with propulsion deficits.

There are limitations associated with this study. Walking conditions in this study were much shorter than previously established training requirements for adaptation to an ankle EXO, (e.g., about 25 minutes [69] to 110 minutes [70]). Here, we investigated the interaction of assistance levels and TLA, so adaptation to the device was neither expected nor relevant for our study purposes. Additionally, to ensure we were assessing EXO × TLA feedback interactions, we did not include a condition with EXO assistance but without feedback of TLA. Based on previous literature, however, we would expect the TLA to decrease, and propulsion to remain unchanged in such a condition [14,15]. However, we still tested a decreased, typical, and increased TLA with all EXO torques allowing a full landscape of possible interactions. Another limitation of this study is that multiple imputation was used for one condition for one participant. Although multiple imputation is generally acceptable, it is possible that this skewed our results given our relatively small sample size [47–49]. Our participants were successful in adjusting their TLA in real-time according to the prescribed target TLA for all 3 target conditions. Although participants did not quite reach the −5° from baseline TLA, the induced changes to peak TLAs were still significantly lower than baseline conditions. Because the participants walked at a fixed speed, we suggest that the increased cadence induced by the shorter steps may have made it difficult to precisely reach the target. Nevertheless, reducing propulsion by reducing TLA while maintaining walking speed is disadvantageous [8–10] and would likely not be employed in clinical rehabilitation or therapy for populations with impairment. This condition was included to capture a complete range of TLA targets to enhance interpretations of the EXO x TLA feedback interactions. Finally, this study included short durations of walking on a treadmill which does not resemble natural walking in the real-world. However, our biomechanical assessment required measurement of ground reaction forces and valid comparisons between conditions required speed to be maintained between conditions, given the impact of walking speed on propulsion [8,71], joint kinetics [72,73], and MoS [32]. These laboratory-based experiments may offer insight into the biomechanics of walking with these interventions that may then be used to guide future work seeking to pair EXOs with real-time kinematic feedback in settings more relevant to natural walking in the community.

## Conclusions

To our knowledge, this is the first study that examined the effects of various levels of EXO assistance and peak TLA targets while walking. The use of visual feedback of TLA increased the propulsive impulse, although this should be implemented with considerations for possible adverse effects on stability. Despite not enhancing propulsion in this study, we showed potential for an ankle EXO to facilitate proximal-to-distal redistribution of positive mechanical work performed by the lower limb at speeds representative of clinical populations. Although this study was conducted in young, unimpaired individuals, our biomechanical analysis serves as a relevant precursor to research and clinical implementation of assistive devices intended to enhance community ambulation in populations with gait impairments.

## Supporting information

S1 Fig Within-TLA condition participant-level EXO effect on propulsive impulse. Difference in propulsive impulse from the 0% BW EXO condition to the 15% and 35% BW EXO conditions within each respective target TLA condition. Minimal detectable change (MDC) of propulsive impulse shown for within-test analysis [74].
(TIF)

S1 Table Positive mechanical work performed by each joint. Positive mechanical work (J/kg) performed over a stride by the biological ankle, EXO, knee, hip, and total limb for each condition. Values reported as mean±SD.
(DOCX)
