# Supplementary figures and images for: Pairing limb posture feedback with an ankle exoskeleton to augment limb propulsion
Source: PLoS One. 2025 Oct 22;20(10):e0335054. doi: 10.1371/journal.pone.0335054 (PMC12543136; doi:10.1371/journal.pone.0335054)

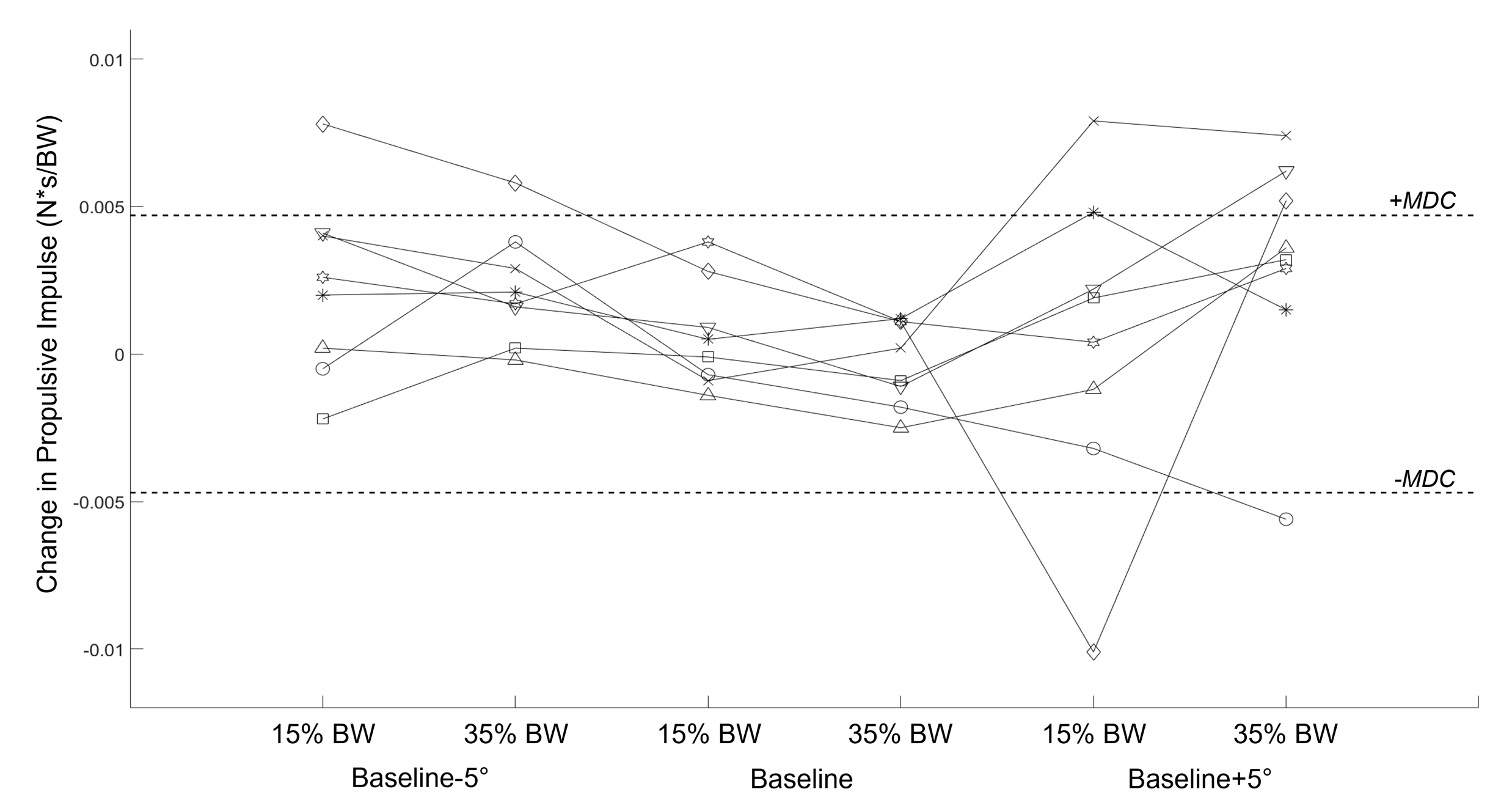

Supplement: S1 Fig — Difference in propulsive impulse from the 0% BW EXO condition to the 15% and 35% BW EXO conditions within each respective target TLA condition. Minimal detectable change (MDC) of propulsive impulse shown for within-test analysis [74]. (TIF) [file pone.0335054.s001.tif]
