# Supplementary material for: Pairing limb posture feedback with an ankle exoskeleton to augment limb propulsion
Source: PLoS One. 2025 Oct 22;20(10):e0335054. doi: 10.1371/journal.pone.0335054 (PMC12543136; doi:10.1371/journal.pone.0335054)
Supplement: S1 Table — Positive mechanical work (J/kg) performed over a stride by the biological ankle, EXO, knee, hip, and total limb for each condition. Values reported as mean±SD. (DOCX) [file pone.0335054.s002.docx]

| **S1 Table. Positive mechanical work performed by each joint.** | | | | | | | | | | |
| --- | --- | --- | --- | --- | --- | --- | --- | --- | --- | --- |
| Positive mechanical work (J/kg) performed over a stride by the biological ankle, EXO, knee, hip, and total limb for each condition. Values reported as mean±SD. | | | | | | | | | | |
|  |  |  |  |  |  |  |  |  |  |  |
| **TLA Condition** | **Typical** | **-5° TLA** | **-5° TLA** | **-5° TLA** | **Baseline** | **Baseline** | **Baseline** | **+5° TLA** | **+5° TLA** | **+5° TLA** |
| **EXO Condition** | **0% BW** | **0% BW** | **15% BW** | **35% BW** | **0% BW** | **15% BW** | **35% BW** | **0% BW** | **15% BW** | **35% BW** |
| **Biological Ankle** | 0.30±0.08 | 0.22±0.07 | 0.30±0.08 | 0.35±0.06 | 0.31±0.06 | 0.37±0.06 | 0.39±0.07 | 0.43±0.14 | 0.47±0.13 | 0.48±0.14 |
| **Ankle EXO** | 0.00±0.00 | 0.00±0.00 | 0.01±0.00 | 0.03±0.01 | 0.00±0.00 | 0.01±0.01 | 0.03±0.01 | 0.00±0.00 | 0.02±0.00 | 0.04±0.01 |
| **Knee** | 0.12±0.03 | 0.11±0.03 | 0.14±0.03 | 0.15±0.05 | 0.14±0.05 | 0.19±0.06 | 0.21±0.07 | 0.19±0.12 | 0.19±0.05 | 0.24±0.09 |
| **Hip** | 0.35±0.08 | 0.42±0.07 | 0.39±0.08 | 0.39±0.06 | 0.36±0.09 | 0.34±0.09 | 0.34±0.09 | 0.35±0.08 | 0.34±0.12 | 0.33±0.11 |
| **Total** | 0.78±0.12 | 0.76±0.08 | 0.84±0.09 | 0.92±0.09 | 0.81±0.13 | 0.92±0.10 | 0.96±0.09 | 0.97±0.11 | 1.02±0.16 | 1.09±0.17 |
